# Constitutive and LPS-stimulated secretome of porcine Vascular Wall-Mesenchymal Stem Cells exerts effects on in vitro endothelial angiogenesis

**DOI:** 10.1186/s12917-019-1873-1

**Published:** 2019-04-27

**Authors:** Chiara Bernardini, Martina Bertocchi, Augusta Zannoni, Roberta Salaroli, Irvin Tubon, Giovanni Dothel, Mercedes Fernandez, Maria Laura Bacci, Laura Calzà, Monica Forni

**Affiliations:** 1 Department of Veterinary Medical Sciences DIMEVET, University of Bologna, Via Tolara di Sopra 50, 40064 Ozzano Emilia, Bologna, Italy; 2 Department of Medical and Surgical Sciences – DIMEC, University of Bologna, Bologna, Italy; 3 Department of Pharmacy and Biotechnology – FaBiT, University of Bologna, Bologna, Italy; 4 Escuela de Enfermeria, Facultad de Ciencias Medicas, Universidad Regional Autónoma de Los Andes UNIANDES, Ambato, EC180150 Ecuador

**Keywords:** Secretome, Vascular Wall-MSCs, Pig animal model, Angiogenesis

## Abstract

**Background:**

MSCs secretome is under investigation as an alternative to whole-cell-based therapies, since it is enriched of bioactive molecules: growth factors, cytokines and chemokines. Taking into account the translational value of the pig model, the leading aim of the present paper was to characterize the secretome of porcine Vascular Wall–Mesenchymal Stem Cells (pVW-MSCs) and its change in presence of LPS stimulation. Moreover, considering the importance of angiogenesis in regenerative mechanisms, we analysed the effect of pVW-MSCs secretome on in vitro angiogenesis.

**Results:**

Our results demonstrated that conditioned medium from unstimulated pVW-MSCs contained high levels of IL-8, GM-CSF, IFN-γ and other immunomodulatory proteins: IL-6 IL-18 IL-4 IL-2 IL-10. LPS modulates pVW-MSCs gene expression and secretome composition, in particular a significant increase of IL-6 and IL-8 was observed; conversely, the amount of GM-CSF, IFN-γ, IL-2, IL-4, IL-10 and IL-18 showed a significant transient decrease with the LPS stimulation. Conditioned medium from unstimulated pVW-MSCs induced in vitro endothelial angiogenesis, which is more evident when the conditioned medium was from LPS stimulated pVW-MSCs.

**Conclusions:**

The lines of evidence here presented shed a light on possible future application of secretome derived by pVW-MSCs on research studies in translational regenerative medicine.

## Background

Increasing evidences have confirmed the dynamic nature of blood vessels, defining the vasculature as a reservoir for a range of multipotent and lineage-restricted progenitor cells, both during embryonic and postnatal life [1–3]. In order to simplify the copious classification, some authors have identified two main classes of multipotent mesenchymogenic populations derived from vessels: microvascular multipotent pericytes [4] and Vascular Wall Mesenchymal Stem Cells (VW-MSCs) [5, 6].

The presence of pro-angiogenic progenitor cells in the arterial wall, including aorta, has been shown in different species including human [7–9], mouse [10] and rat [11]. Many similarities between human and porcine Mesenchymal Stem Cells (MSCs) has been demonstrated [12–15] confirming the swine as an excellent translational model also in the field of regenerative medicine. Furthermore, we have established that porcine Aortic Vascular Precursor Cells (pAVPCs) possess pro-angiogenic features for their ability to differentiate toward the endothelial phenotype and also for their pericyte-like proprieties [16]. Taking into account all these observations we could classify them as porcine Vascular Wall–MSCs (pVW-MSCs) [6].

Regardless their vascular origin, vascular stem cells exhibit an intrinsic pro-angiogenic attitude by different features: the capacity of differentiating towards endothelial phenotype, the ability to sustain the formation of a capillary network [9, 11, 17, 18] and the secretion of angiogenic factors including bFGF, VEGF, TGF-β, PDGF, IL-6, and IL-8 [19]. Overall, vascular stem cells can reasonably be considered for their application in the field of regenerative medicine. To date clinical treatments with MSCs are based on their transplantation [20] but the main drawback associated with these current therapies is represented by bio-distribution of injected cells. Indeed, whole-cell systemic treatment is mostly applied for those disorders in which the site for MSCs putative therapeutic activity is where cells remain entrapped once in the system, as in the alveolar microcirculation [21]. Some reports ascribed MSCs regenerative ability to the paracrine effects exerted by the MSCs-derived mediators. Accordingly, MSCs secretome is under investigation as an alternative to whole-cell-based therapies [22]. MSCs secretome is enriched of bioactive factors, such as growth factors, cytokines and chemokines known to modulate immune response, inhibit apoptosis and fibrosis and enhance angiogenesis by stimulating differentiation of tissue resident progenitor cells [23, 24]. The secretome composition may vary widely depending on the source tissue and can be modulated by different stimuli. Among vascular stem cells, microvascular pericyte release a heterogenous secretome in response to high glucose concentrations [25], or inflammatory interleukins [26]. Moreover, LPS binding of Toll-Like Receptor4 (TLR4) on perycites induces the secretion of several pleiotropic cytokines such as IL-6, IL-8 and chemokines CXCL10, CCL2 [26].

Taking into account the translational value of the pig model and considering our previous data describing the marked angiogenic properties of pVW-MSCs, the leading aim of the present work was to characterize pVW-MSCs secretome. Therefore, we studied the change of pVW-MSCs secretome induced by LPS stimulation, after checked the presence of TLR4 on pVW-MSCs. Furthermore, in order to address a possible application of pVW-MSCs secretome in the study of reparative medicine, we analysed the effect of conditioned medium by unstimulated or LPS-stimulated pVW-MSCs on in vitro angiogenesis.

## Results

### TLR4 expression in pVW-MSCs

First of all, we assessed the TLR4 receptor expression by flow cytometry both in not-fixed and not-permeabilized cells than in fixed and permeabilized pVW-MSCs.

**Fig. 1:**
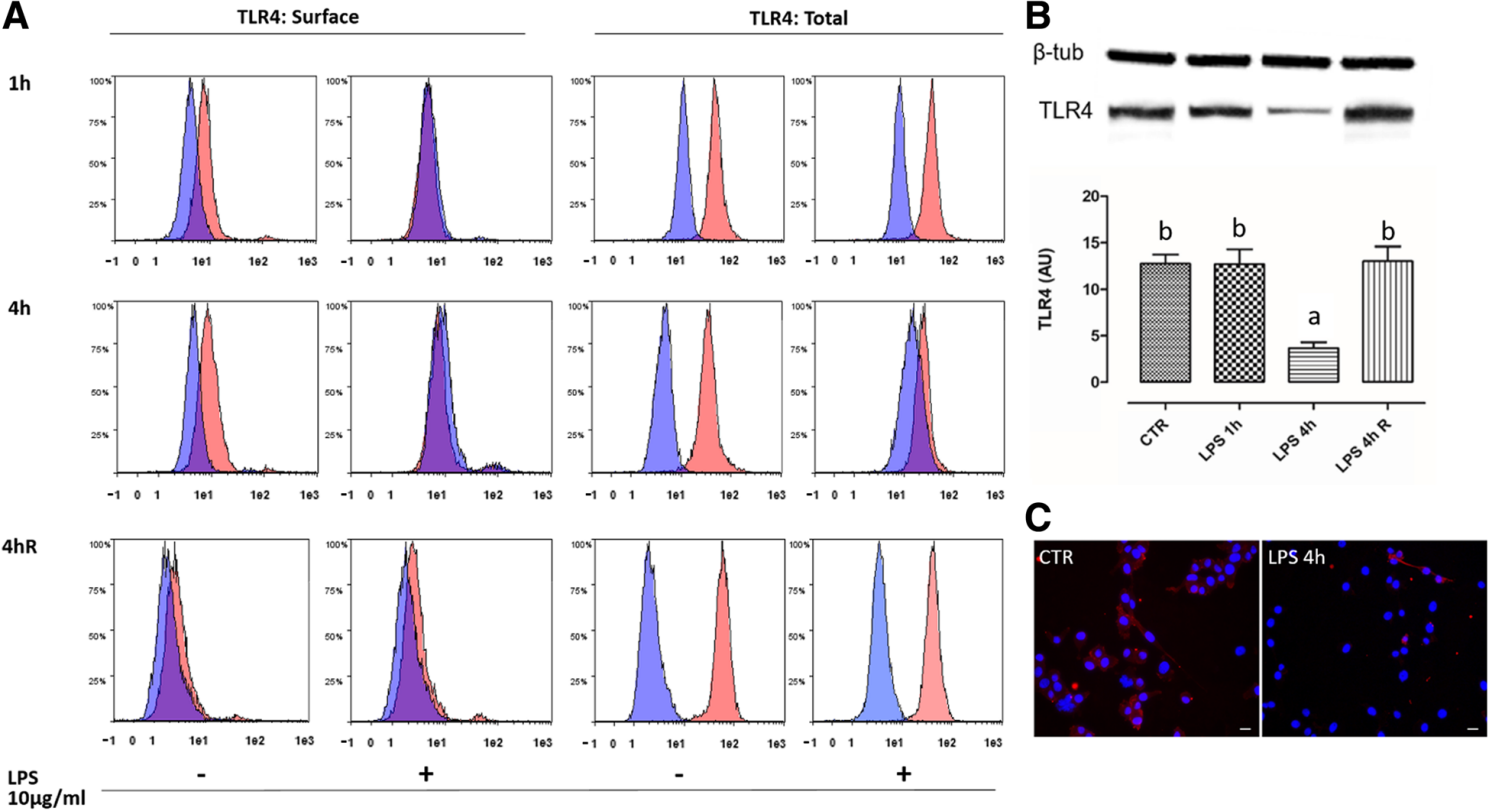
TLR4 expression in pVW-MSCs cultured with or without LPS (10 μg/ml) for 1 and 4 h and after additional 24 h of recovery after LPS removal (4hR). **a***:* flow cytometry analysis were performed in not fixed and not permeabilized cells for TLR4 surface expression determination (TLR4: Surface) and in fixed and permeabilized cells to measure the overall TLR4 amount (TLR4: Total). Red histograms: stained cells; blue histograms: control cells. **b**: representative Western Blot of TLR4 and housekeeping β-tubulin and relative quantification were presented. **c**: representative images of TLR4 immunostaining of pVW-MSCs cultured with or without LPS (10 μg/ml) for 4 h. pVW-MSCs nuclei were stained with Hoechst 33258 (blue). Scale bar = 10 μm. Data shown represent the mean ± SD of three biological replicates, each experiment is repeated three times. Data were analysed using one-way ANOVA followed by the Tukey’s post hoc comparison test. Different letters above the bars indicate significant differences (*p* < 0.05). (AU = Arbitrary Units)

Cytofluorimetric analysis showed that TLR4 was expressed in the pVW-MSCs cells at each experimental time point. In particular, the untreated cells showed an high level (97.91 ± 2.34%) of total TLR4 in nearly the totality of cell population as shown in fixed and permeabilized samples (Fig. 1a). Differently, surface TLR4 expression was markedly lower (10.43 ± 1.77%) (Fig. 1a). After 1 h of LPS stimulation, TLR4 signal disappeared completely in the surface even if the total expression levels remained essentially unchanged (97.01 ± 3.35%). After 4 h of LPS stimulation, total TLR4, that is the sum of surface and intracellular receptor, expression markedly decreased not only on the surface but also as a whole (CTR 98.47 ± 2.67%, LPS 10 μg/ml 5.93 ± 1.11%). A recovery period of 24 h after 4 h of LPS stimulation re-established the level of total TLR4 expression (99.58 ± 2.63%). Western blot and (Fig. 1b) confirmed the expression of TLR4 in pVW-MSCs and its decrease after 4 h of LPS stimulation. The immunofluorescence analysis showed a drastic reduction of positive cells after LPS treatment (98 ± 2 vs 3 ± 1 95%) (Fig. 1c).

### pVW-MSCs characterization after LPS treatment

**Fig. 2:**
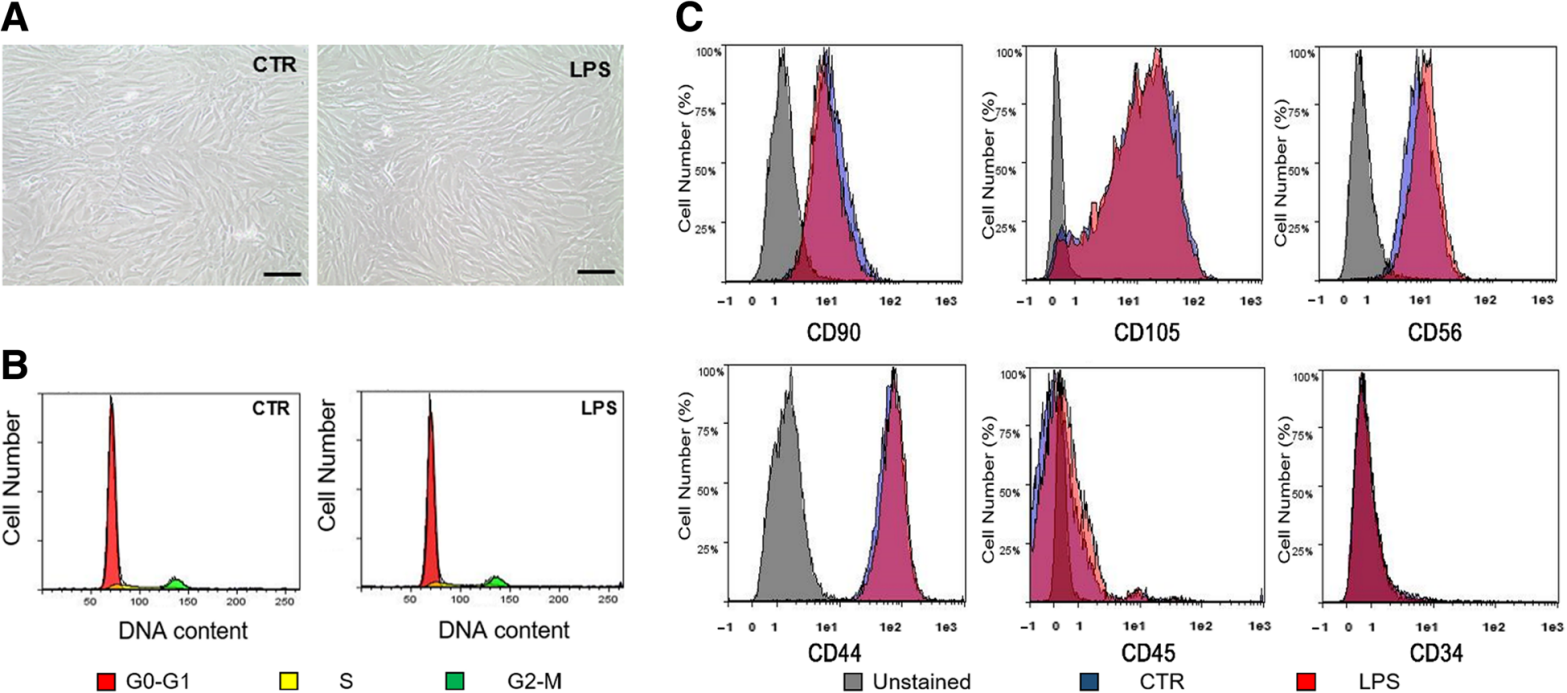
Phenotypic analysis of LPS treated pVW-MSCs. Cells were cultured with or without LPS (10 μg/ml) for 4 h. **a**: representative images of pVW-MSCs morphology. Scale bar = 100 μm. **b**: cell cycle analysis: red: cells in G0-G1 phase, yellow: cells in S phase, green: cells in G2-M phase. **c**: immunophenotyping: flow cytometry analysis for mesenchymal markers: grey histograms: unstained cells, blue histograms: control cells, red histograms: LPS-treated cells. Results are representative of three biological replicates; each experiment is repeated three times

**Fig. 3:**
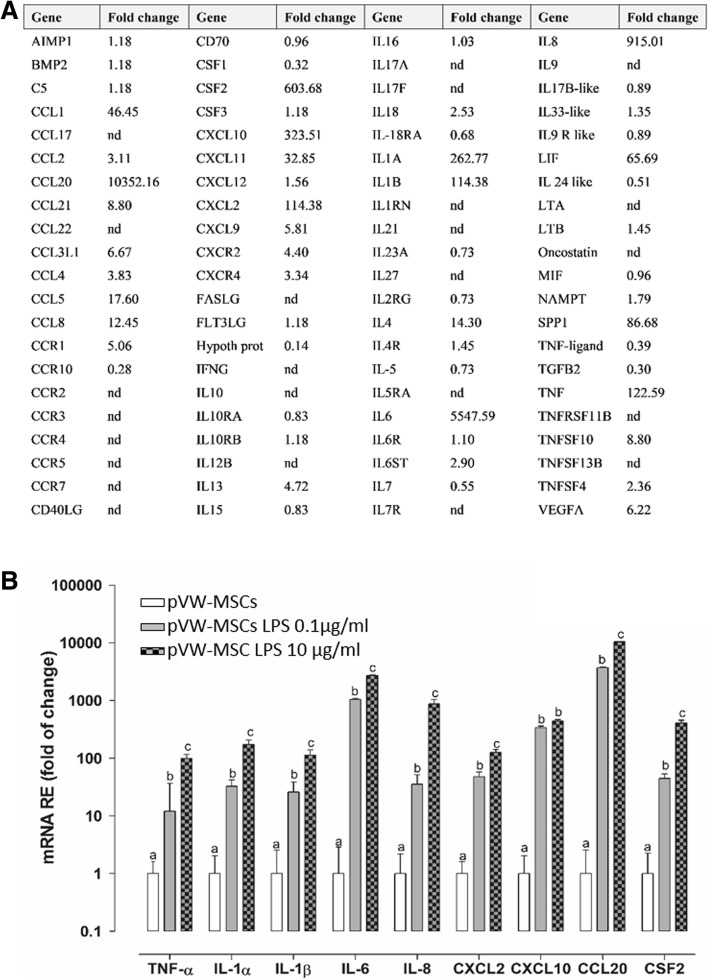
Transcriptional profile of cytokine and chemokine in pVW-MSCs after LPS treatment **a**: cytokines and chemokines relative expression evaluated by RT^2^ Profiler™ PCR Array Pig Cytokines & Chemokines in pVW-MSCs cultured with LPS (10 μg/ml) for 4 h. **b**: qRT-PCR analysis of selected genes in pVW-MSCs cultured in control condition and with LPS 0.1 μg/ml or10 μg/ml for 4 h. Data represent the mean ± the range of relative expression of three biological replicates, each experiment is repeated two times. Data were analysed using one-way ANOVA followed by the Tukey’s post hoc comparison test. Different letters above the bars indicate significant differences (*p* < 0.05). The relative mRNA expression (fold of change) was calculated in relation to control cells using the 2^-ΔΔct^ method. nd = not detectable

No sign of toxicity was evidenced by MTT test after LPS treatment at the doses and times used (data not shown). Therefore, we assessed whether LPS treatment could cause any change of pVW-MSCs phenotype. pVW-MSCs showed a thin spindle-shaped morphology typical for mesenchymal and vascular wall mesenchymal cells; LPS treatment (10 μg/ml 4 h) did not alter this characteristic cellular morphology (Fig. 2a). pVW-MSCs showed a standard cell cycle characterizing diploid cells (CTR: phase G0-G1 84.92 ± 5%, phase S 8.76 ± 0,3%, phase G2-M 6.32 ± 0.3%) that remained unchanged after LPS treatment (LPS: phase G0-G1 84.22 ± 5%, phase S 9.04 ± 0.8%, phase G2-M 6.74 ± 0.3%) (Fig. 2b). Cytofluorimetric analysis (Fig. 2c) confirmed that LPS treatment did not alter pVW-MSCs positivity for major MSC markers: CD105 (CTR 96.8 ± 0.4% vs LPS 96.3 ± 0.2%), CD90 (CTR 96.2 ± 0.3% vs LPS 96.5 ± 0.1%), CD56 (CTR 98.4 ± 0.3% vs LPS 98.8 ± 0.3%), CD44 (CTR 99.4 ± 0.2% vs LPS 99.4 ± 0.1%). Negative immunoreactivity for hematopoietic CD45 (CTR 2.6 ± 0.7% vs LPS 2.7 ± 0.6%) and endothelial markers CD34 (CTR 2.7 ± 0.9% vs LPS 1.7 ± 0.4%) remained unchanged. In order to investigate the possible effect of LPS on gene expression of potential secreted molecules we used a panel of porcine cytokines and chemokines genes. After LPS treatment, pVW-MSCs showed an altered gene expression profile: among the 84 genes of the RT^2^ Profiler™ PCR Array Pig Cytokines & Chemokines, 30 genes were upregulated (fold of change > 2). Among these genes, 9 were expressed more than 100-fold (Fig. 3a).

The array validation of selected genes (fold of change > 100), confirmed the gene expression profile determined by the array analysis. The lowest dose too (LPS 0.1 μg/ml) was effective in stimulating the expression of the same selected genes (Fig. 3b).

### Cytokines and chemokines presence in pVW-MSCs conditioned media and their alteration induced by LPS treatment

**Fig. 4:**
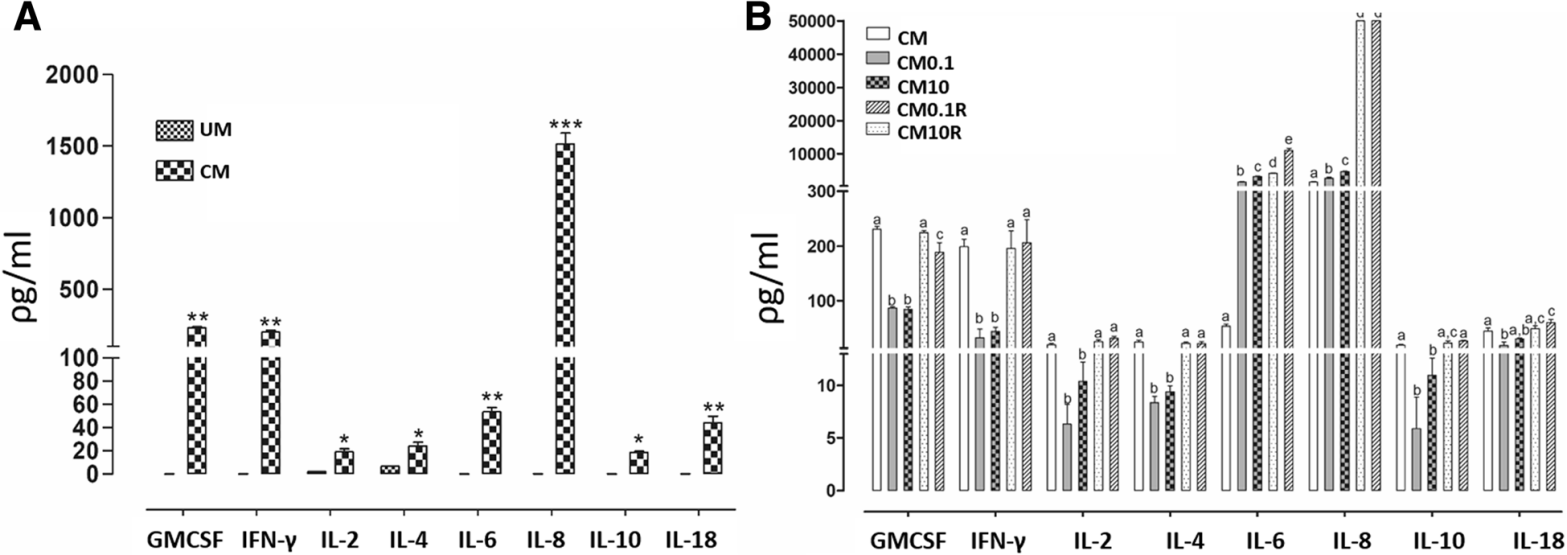
Cytokines in pVW-MSCs conditionate medium. **a**: the concentration of cytokines/chemokines was measured in conditioned medium obtained from unstimulated pVW-MSCs (CM), unconditioned medium (UM). Data represent the mean ± SD of three biological replicates, each experiment was repeated three times. Data were analysed using Student’s *t*-test. Significant differences are indicated by *p < 0.05, **p < 0.01, ***p < 0.001. **b***:* the concentration of cytokines was measured in conditioned medium obtained from pVW-MSCs treated LPS with LPS 0.1 μg/ml (CM 0.1), 10 μg/ml (CM 10) and from recovery time (CM0.1R; CM10R). Data represent three biological replicates, each experiment was repeated three times and represent the mean ± SD. Data were analysed using one-way ANOVA followed by the Tukey’s post hoc comparison test. Different letters above the bars indicate significant differences (*p* < 0.05)

CM from unstimulated pVW-MSCs contained high levels of and IL-8 (1514 ± 77.1 pg/ml), GM-CSF (230 ± 5.3 pg/ml), IFN-γ (199 ± 13.8 pg/ml); other immunomodulatory proteins were also detected: IL-6 (53.5 ± 3.8 pg/ml), IL-18 (44 ± 5.6 pg/ml). IL-4 (24.07 ± 3.38 pg/ml), IL-2 (19.16 ± 2.38 pg/ml), IL-10 (18.7 ± 0.9 pg/mL) (Fig. 4a).

CM0.1 and CM10, derived from LPS stimulated pVW-MSCs, showed a significant increase of IL-6 and IL-8; this increase was maintained also at the end of recovery time with IL-8 showing an additional noticeable increase (CM0.1R and CM10R) (Fig. 4b).

Conversely, the amount of GM-CSF, IFN-γ, IL-2, IL-4, IL-10 and IL-18 showed a significant transient decrease with the LPS stimulation (CM0.1 and CM10) and restored the basal levels after recovery time (CM0.1R and CM10R) (Fig. 4b).

IL-1α, IL-1β, IL-1ra, IL-12 and TNF were undetectable in all CMs (data not shown).

### Effect of conditioned media on endothelial cell angiogenesis

**Fig. 5:**
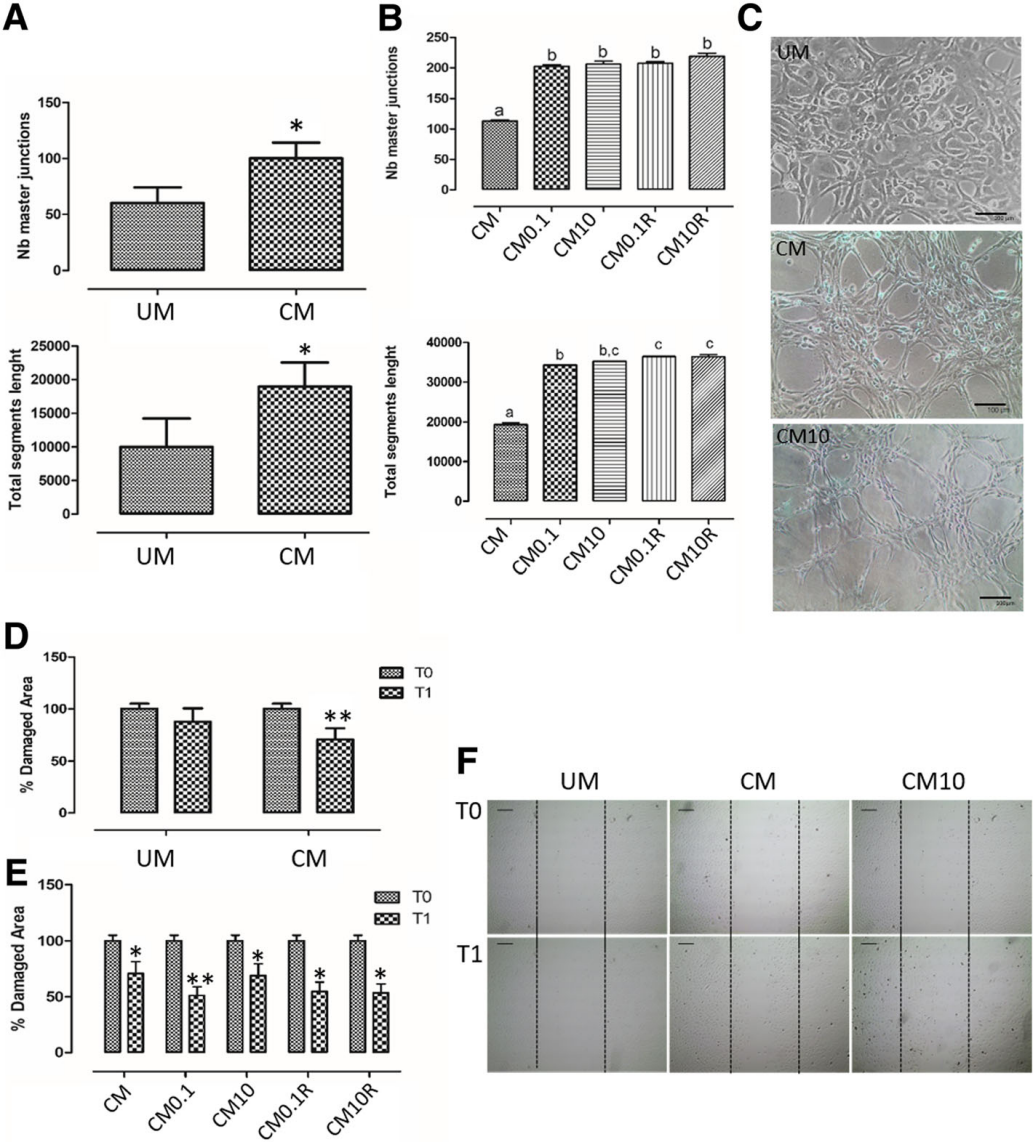
Effect of pVW-MSCs conditioned medium on endothelial cells angiogenesis. **a***:* the presence of pVW-MSCs conditioned medium (CM) induced endothelial cells to form a capillary like network compared to unconditioned medium (UM). **b***:* pAECs cultured in extracellular matrix coating were exposed to conditioned medium obtained from unstimulated pVW-MSCs (CM) or 0.1 and 10 μg/ml LPS treated pVW-MSCs (CM 0.1; CM 10) and from recovery (CM0.1R; CM10R). **c**: representative images of pAECs in vitro angiogenesis in the presence of UM, CM, CM10, CM10. Scale bar = 100 μm. **d, e, f***:* the effect of conditioned medium on endothelial cells migratory capacity was demonstrated by scratch test. Confluent pAECs were scratched (T0) and exposed to UM and CM, CM0.1, CM10, CM0.1R, CM10R for 24 h (T1). All data shown represent the mean ± SD of three biological replicates, each experiment is repeated two times. Data (Fig. a, d, e) were analysed using Student’s *t*-test between different treatment (UM vs CM) or different time (T0 vs T1) for each treatment*.* Significant differences are indicated by *(*p* < 0.05) and **(*p* < 0.01). Data (Fig. b) was analysed using one-way ANOVA followed by the Tukey’s post hoc comparison test, different letters above the bars indicate significant differences (*p* < 0.05)

The addition of CM from unstimulated pVW-MSCs to the endothelial medium stimulated pAECs to form a better structured capillary-like network (Fig. 5a, c) compared to the addition of PGM unconditioned medium (UM) (Fig. 5a, c).

CM0.1 and CM10, derived by LPS-stimulated pVW-MSCs, showed a stronger capacity to induce tube formation compared to the medium from the unstimulated pVW-MSCs (CM) (Fig. 5b, c).

CM0.1R and CM10R also exhibited high capacity of inducing capillary-like network (Fig. 5b).

CMs effect on endothelial cells migration was demonstrated by scratch test. After 24 h from scratch (T1) unstimulated pVW-MSCs CM induced a significant decrease of the damaged area compared to PGM unconditioned medium (UM) (Fig. 5d-f). Besides, CM0.1, CM10, CM0.1 R and CM10 R were able to increase endothelial migration reducing damaged area (Fig. 5e-f).

## Discussion

Over the last decade, a remarkable interest of the scientific community aroused after the discovery of a heterogeneous population of stem cells residing in both microvascular and large vessel, which are involved in the regulation of the vascular physiological homeostasis [5, 6, 27, 28]. Among the stem cells resident in large vessels, those isolated from the aorta showed great pro-angiogenic attitudes [9, 29, 30]. As swine is considered an excellent model for translational purpose, especially in the cardiovascular research area [31–33], we described a new method to obtain vascular precursor cells from pig thoracic aorta: pVW-MSCs [15, 16]. These cells show mesenchymal properties and the ability to differentiate in all the components of a functional vessel. Furthermore, pVW-MSCs sustain capillary-like tube formation in vitro, showing pericyte-like properties, while environmental stressors [34] impaired this ability.

In the present paper, we focus attention on pVW-MSCs secretome. Considering that LPS is the most studied pericyte-activating molecules in vitro we first verified the TLR4 presence in pVW-MSCs. In fact the presence and role of TLR4 in adult stem cells are currently under debate [35, 36], in particular, TLR4 expression in MSCs. In this regard, Chen and colleagues [37] determined low levels of TLR4 in human Umbelical Cord-MSCs, conversely, TLR4 is highly expressed in human adipose MSCs [38]. Moreover, Raicevic et al. [39] did not detect TLR4 on human Wharton’s Jelly-derived MSCs. Our data showed not only TLR4 presence in pVW-MSC but also the LPS ability to modulate its expression. In particular, we detected a decrease of the surface fraction of TLR4 induced by LPS stimulation, while the total amount did not change. The persistence of LPS stimulus resulted in a reduction of the total amount of the receptor, which is restored after a recovery period of 24 h. Therefore, our results show the expression of this ancestral receptor on pVW-MSCs and this is in agreement with previous studies on TLR4 expression in human multipotent pericytes [40].

Upon these bases, we used LPS as a priming stimulus to investigate pVW-MSCs secretome in mild challenging conditions. In fact, LPS at the concentrations and time used in the present paper did not affect pVW-MSCs morphology, viability, cell cycle and immunophenotype.

However, pVW-MSCs gene expression is markedly altered. LPS evoked a cytokine response as shown by the high level of TNF-α, IL-6, IL-8, IL-1α, IL-1β along with an increase of other immunomodulatory molecules (CXCL2, CXCL10 CCL20, CSF2). These results are in line with results obtained with MSCs derived from different sources, including multipotent microvascular human pericytes, (16), human adipose-derived stem cells (29), human bone marrow-derived MSCs [37, 40–42].

The analysis of the presence of cytokines and chemokines in unstimulated pVW-MSCs conditioned medium showed for the first time the presence of molecules with strong immunomodulatory properties such as GM-CSF and IFN-γ, as also reported for cultured MSCs [43]. Considerable levels of IL-8 and IL-6 were also detected in agreement with what evidenced in conditioned medium of other tissue-specific stem cells such as Wharton’s Jelly-derived MSCs [44] and endometrial MSCs [45].

The secretome composition changed after pVW-MSC challenge with LPS. IL-6 and IL-8, dramatically increase conversely, GM-CSF, IFN-γ, IL-2, IL-4, IL-10 and IL-18 levels decreased. Nevertheless, the basal levels of analysed molecules are restored after the 24-h recovery time, except for IL-6 and IL-8, which kept increasing.

Finally, TNF-α, IL-1α, IL-1β are not detected in the culture medium of pVW-MSCs in any of the experimental conditions tested, although gene expression of these cytokines was increased by LPS. Therefore further investigations are warranted to clarify the specific regulation of these cytokines.

An increasing number of paper showed that MSCs can induce angiogenesis through paracrine effects [46, 47] in the present paper we demonstrated the effects of unstimulated pVW-MSCs secretome on endothelial cell angiogenesis. In fact, the addition of pVW-MSCs conditioned medium induced in vitro angiogenesis and endothelial cell migration, confirming high pro-angiogenic effect not only exerted by the cells, but also by molecules secreted. Overall in the present paper we demonstrated the paracrine properties of pVW-MSCs in line with those described for MSC [48].

Priming pVW-MSCs with LPS at different concentrations enhanced both pro-angiogenic effect and the capacity to induce endothelial cell migration. Moreover, similar effects are obtained by the conditioned medium of pVW-MSCs after 24 h of recovery from the LPS stimulus.

This evident in vitro effect on endothelial cells could be reasonably ascribed to the endothelial CXC receptor 2 (CXCR2) activation caused by the observed high levels of IL-8 [49]. However, we can not rule out that other important components present in the conditioned medium of pVW-MSCs may play a role in the induction of angiogenesis. In fact, it was recently demonstrated that MSCs could release exosomes that transfer miRNAs to endothelial cells promoting angiogenesis [50]. Therefore, further studies are necessary to investigate the presence of exosomes, microvescicles and miRNAs in pVW-MSCs conditioned medium.

## Conclusions

Overall, our results demonstrated for the first time the presence of powerful regulatory molecules secreted by pVW-MSCs. The composition of secretome is altered by LPS challenge and some of these differences are kept even after a recovery time. In particular, IL-6 and IL-8 seems strongly regulated. The pVW-MSCs secretome, both unstimulated and LPS induced, is active in the modulation of in vitro angiogenesis.

Although our results are limited to an in vitro approach, this is a fundamental step toward better-designed in vivo experiments, according to commonly-accepted 3Rs rules (Replacement, Reduction and Refinement) [51, 52] for more ethical use of animals in experimental testing and the European Directive on the protection of animals used for scientific purposes (Directive 2010/63/EU) [53]. Therefore the lines of evidence here presented shed a light on possible future application of secretome derived by pVW-MSCs on research studies in translational regenerative medicine.

## Methods

### Chemicals and reagents

Heat inactivated FBS (fetal bovine serum), antibiotic–antimycotic, Dulbecco’s phosphate buffered saline (DPBS), high glucose (hg) DMEM, M199, Endothelial Cell Growth Medium (ECM) and Geltrex™ LDEV-Free Reduced Growth Factor Basement Membrane Matrix and NuPage 4–12% bis-Tris Gel, were purchased from Gibco-Life Technologies (Carlsbad CA, USA). Trypsin–EDTA solution1X, Dimethyl sulphoxide (DMSO), lipopolysaccharide (LPS) (*E. coli* 055:B5), Protein Assay Kit TP0300, In Vitro Toxicology Assay Kit and Cell Growth Determination Kit MTT based were purchased from Sigma-Aldrich (St. Louis, MO, USA). Pericyte Growth Medium was purchased from Promocell (Heidelberg, Germany). NucleoSpin RNA kit was purchased from Macherey-Nagel GmbH & Co. KG (Düren, Germany) RT^2^ strand kit, RT^2^ Sybr green fluor qPCR master mix were from Qiagen (Hilden, Germany). Porcine Cytokine/Chemokine Magnetic Bead Panel kit, Milliplex Map Kit EMD was purchased by Millipore Corporation (Billerica, MA, USA). Super Signal West Pico Chemiluminescent Substrate was from Pierce Biotechnology, Inc. (Rockford, IL, USA).

### Cell culture

pVW-MSCs were isolated from female 3-mo-old pigs (Large White) euthanized for other experimental purposes, following the published methods previously described [15], to generate three primary cell culture replicates. All procedures on pigs were reviewed and approved in advance by the Ethical Committee of the University of Bologna (Bologna, Italy) and were then approved by the Italian Ministry of Health (Protocol number n.43-IX/9 all.37; 20/11/2012)*.* Briefly, cells were isolated from the media layer of the aortas through an enzymatic digestion and cultured overnight in high glucose (hg) DMEM 10% FBS and 10X antibiotic-antimycotic (hgDMEM-10X) in a 5% CO_2_ incubator at 38.5 °C. The day after the culture medium was replaced by hgDMEM + 10% FBS (GIBCO) + 1X antibiotic-antimycotic (GIBCO) (hgDMEM-1X). After 3 days cells were serum starved overnight and then cultured in hgDMEM:M199 (GIBCO) (1:1) 10% FBS 1X antibiotic-antimycotic (DM medium). Then cells were trypsinized, grown and expanded not beyond till passage (P) 6 in Pericyte Growth Medium (PGM – Promocell, Heidelberg, Germany). All the experiments described in this paper were performed with cells at the third passage (P3), cultured in Pericyte Growth Medium (PGM).

The LPS treatments were performed once reached 80% confluence as reported in the specific sections, cells viability after the treatments was determined by MTT test (In vitro Toxicology assay kit, MTT based TOX1-1KT, Sigma). Porcine Aortic Endothelial Cells (pAECs) were isolated by thoracic aorta of animals slaughtered at a local slaughterhouse and cultured as previously described [54].

### Preparation of conditioned media

Once established that the treatment with LPS for 4 h had no effects on cell cycle, nor on the mesenchymal phenotype, we proceeded with the preparation of the conditioned media. pVW-MSCs from three primary cell cultures, were plated in a 24-multi well plate at a concentration of 3 × 10^4^ cells/well. The day after, the cells were washed three times with DPBS, and then fresh medium with LPS (0; 0.1 and 10 μg/ml) was added. After 4 h, Conditioned Medium (CM) from pVW-MSCs treated with 0.1 μg/ml LPS (CM 0.1), 10 μg/ml LPS (CM 10) or without LPS (CM) was collected.

An additional experiment was conducted to produce CM from recovery (CM0.1R; CM10R): after 4 h of LPS treatment, the cells were washed three times with DPBS, then fresh medium (PGM) was added and the cells were incubated for 24 h (R recovery time).

All collected media were centrifuged at 800 x g for 10 min, filtered through a 0.20-μm syringe filter and immediately frozen in liquid nitrogen.

Unconditioned medium (UM), namely the Pericyte Growth Medium, was used as control.

### pVW-MSCs immunophenotyping and DNA content by flow cytometry

All cytofluorimetric assays were performed with the employment of MacsQuant10 Cytometer and analysed with Flowlogic™ software (Miltenyi Biotec, Bergisch Gladbach, Germany).

LPS was used only as a priming to stimulate secretome change, therefore alteration of pVW-MSCs immunophenotype and/or cell cycle were evaluated. Briefly, CD90, CD105, CD56, CD44, CD45 and CD34 expression were evaluated in LPS-treated and in control cells as previously indicated [15, 16].

For cell cycle analysis pVW-MSCs were centrifuged at 500 x g for 10 min and counted by a hemocytometer. Appropriate volume of 70% ice-cold ethanol (1 ml/10^6^ cells) was added drop-by-drop to cellular pellet vortexing. Single cell suspension was than fixed overnight at + 4 °C. The day after, cells were washed in PBS and incubated for 20 min in the dark with the Staining Solution [PI 50 μg/ml (Miltenyi Biotec, Bergisch Gladbach, Germany), RNAseA/T1 Mix 100 Kunitz/ml (Thermo Scientific, Waltham, MA, USA)]. For cell cycle analysis, Dean-Jett-Fox Univariate Model was applied.

### Determination of TLR4 expression on pVW-MSCs

The determination of TLR4 expression was performed at the highest dose utilized (10 μg/ml) and after 1 and 4 h of exposure. An additional time point was represented by cells exposed to LPS for 4 h and let to recover in standard culture conditions (PGM) for other 24 h (R).

5 × 10^5^ pVW-MSCs were seeded in T25 flasks and LPS was added at 80% of confluence.

At each experimental time point, the cells were harvested and analyzed by flow cytometry, western blot and immunocytochemistry.

TLR4 was determined by flow cytometry in fresh (not fixed and not permeabilized cells) and in fixed/permeabilized cells to distinguish the amount of TLR4 expressed on the surface from the total one, that is the amount of surface receptor and amount of intracellular receptor. TLR4 determination was carried out by incubating 1 × 10^6^/100 μl cells with a specific antibody (1:50, Mouse anti TLR4, NB100–56566 Novus Biologicals Europe) for 60 min at 4 °C in the dark. After incubation, cells were washed twice with DPBS and incubated with secondary antibody (1:50, Anti-Mouse FITC-conjugated F4143, Sigma Aldrich) for 40 min at 4 °C in the dark. At the same time an equal amount of cells for each sample and each time point was fixed and permeabilized with FIX & PERM Cell Permeabilization Kit (Thermo Scientific, Waltham, MA, USA) which allows fixation and permeabilization of cells but, at the same time, leaves their morphological scatter characteristics intact and so it is suitable to accurately identify both superficial and also intracellular markers.

For Western Blot analysis cells were lysed in SDS solution (Tris–HCl 50 mM pH 6.8; SDS 2%; glycerol 5%). Total protein amount was determined by Peterson’s Modification of Lowry Method using a Protein Assay Kit (Sigma). 20 μg of proteins were separated on 4–12% bis-Tris Gel for 45 min at 165 V. Proteins were then electrophoretically transferred onto a nitrocellulose membrane by Turbo Blot System (Bio-Rad Laboratories Inc., Hercules, CA, USA).

Non-specific binding on nitrocellulose membranes was blocked with 5% milk powder in PBS-T20 (Phosphate Buffer Saline-0.1% Tween-20) for 1 h at room temperature. Then, the membranes were probed for the anti-TLR4 antibody (1:500) and an anti-β-tubulin antibody (1:500 of anti β-tubulin MA1–19162, Thermo Fisher Scientific, Rockford, IL, USA). The membranes were developed using a chemiluminescent kit according to the manufacturer’s instructions (Clarity Biorad). The intensity of the luminescent signal of the resultant specific bands was acquired by Chemidoc Instrument using Inage Lab Software (Bio-Rad). The relative protein content (TLR4/β-tubulin) was expressed as arbitrary units (AUs).

In order to assess TLR4 by indirect immunofluorescence, pVW-MSCs were seeded on 8-well slide chamber (BD Falcon Bedford, MA, USA) at a concentration of ~ 3 × 10^4^ cells/well and stimulated with LPS as indicated above. At all experimental time points, pVW-MSCs were washed in PBS and fixed in 4% paraformaldehyde for 20 min at RT. Subsequently, fixed cells were permeabilized with 0.2% Triton-X100 in PBS for 3 min, washed in PBS and blocked by 5% FBS in PBS (blocking solution) for 1 h at RT. The incubation with the anti TLR4 antibody diluted 1:50 in blocking solution was performed for 36 h at 4 °C in humid atmosphere. Then cells were rinsed in PBS and incubated with a fluorochrome-labeled secondary antibody (Goat anti-mouse AlexaFluor 594, A11032, Thermo Fischer Scientific) diluted 1:400 added with 0.1 μg/mL Hoechst 33258 for nuclei staining in PBS for 1 h at RT. Negative controls were carried out by omitting primary antibody. From each slide, at least 6 photomicrographs were acquired using an Eclipse E600 epifluorescence microscope equipped with a Nikon digital camera and the ACT-2 U software for image capturing (Nikon, Tokyo, Japan). Images were analyzed by counting a minimum of 200 cells in order to evaluate the number of positive cells.

### Cytokines & chemokines analysis by RT^2^ assay and real-time PCR

Total RNA from pVW-MSCs treated with or without LPS 10 μg/ml, for 4 h was purified as previously described [16] and quantified with nanospectrophotometer (Denovix, Wilmington, DE, USA). 1 μg of RNA was retro-transcribed using RT^2^ First Strand Kit (Qiagen, Hilden, Germany) following the manufacturer’s instructions, in a 20 μl final volume to obtain cDNA. RT^2^ Profiler™ PCR Array Pig Cytokines & Chemokines (Cat. No. PASS-150Z, Qiagen, Hilden, Germany) was performed according to the manufacturer’s instructions, using CFX 96 Touch (Bio-Rad Laboratories, Hercules, CA) on cDNA pool of the three biological replicates.

In order to validate the Array results, qPCR real time was performed for those genes up-regulated more than 100 times (TNFα; IL1-α; IL-1β; IL-6; IL-8; CXCL2; CXCL10; CCL20; CSF2) respect to the control. A master mix of the following reaction components was prepared in a 25 μl final volume by using RT^2^ SYBR green master mix and RT2 Primer Assay (RT^2^ qPCR Primer Assay for Pig TNF-α, IL1-α, IL1-β, IL-6, IL-8, CXCL2, CXCL10, CCL20, CSF2; Cat. No. PPS00426A, PPS00434A; PPS00461B; PPS00991A; PPS00237A; PPS00036B; PPS01275A; PPS01329A, PPS00543A respectively, Qiagen). cDNA was added to the master mix in a ratio of 1:25 in 25 μl total reaction value and amplified under the following conditions:10 min at 95 °C, 40 cycles at 95 °C for 15 s and at 60 °C for 30 s, followed by a melting step from 55 °C to 95 °C (80 cycles of 0.5 °C increase/cycle). Gene expression was evaluated using the ΔCt method (reference gene Ct – interest gene Ct). Genes up-regulated more than 100 times were studied for further treatments with at a lower concentration of LPS (0.1 μg/ml, 4 h).

The relative mRNA expression of the tested genes was calculated in relation to the control cells using the 2^-ΔΔct^ method [55].

### Multiparametric cytokines and chemokines quantification

Cytokines and chemokines were quantified in conditioned medium (CM) by using the Porcine Cytokine/Chemokine Magnetic Bead Panel kit (PCYTMAG Millipore Corporation, Billerica, MA, USA) including GM-CSF, IFN-γ, IL-1α, IL-1β, IL-1RA, IL-2, IL-4, IL-6, IL-8, IL-10, IL-12, IL-18 and TNF-α, following the manufacturer’s instructions. Luminex xMAP bead-based multiplex immunoassay technology and MAGPIX instrument provided with xPONENT 4.2 software were used (Luminex, Austin, TX, USA).

### Evaluation of the effect of conditioned medium on endothelial cells angiogenesis

To evaluate the effects of conditioned medium from pVW-MSCs on endothelial cells angiogenesis, 30% of different CM (UM, CM0, CM0.1, CM10, CM0.1R,CM10R) was added to the endothelial medium and pAECs were tested for in vitro endothelial angiogenesis assays (tube formation assay and scratch test).

To perform in vitro endothelial tube formation assay, 5 × 10^4^/well pAECs were cultured in 8-well slide chambers (BD Falcon Bedford, MA USA) coated with undiluted soluble form of basement membrane extracted from murine Engelbreth-Holm-Swarm (EHS) tumors [(Geltrex™ LDEV-Free Reduced Growth Factor Basement Membrane Matrix (Catalog number: A1413201Thermo Fisher)], as previously reported [15, 16]. Extracellular matrix coating was carried out for 3 h in a humidified incubator, at 38.5 °C, 5% CO_2_. At the end of experimental time (24 h), the images, acquired using a Nikon epifluorescence microscope equipped with digital camera (Nikon, Yokohama, Japan), were analysed by open software Image J 64 to measure the sum of length of the segments in the analyzed area (Tot. segments length) and the number of master junctions in the analyzed area (Nb master junction).

To perform endothelial cell migration assay (Scratch test), a wound was scratched with a pipette tip, in the confluent pAECs layer in a 24-wells plate. Then, cells were washed with DPBS to remove debries and complete medium added with 30% of different CM was added. Microscopic bright field pictures and three measurements of the damaged areas were taken immediately after the scratches (T0) and at the end of the experiment (24 h, T1). Images were acquired using a Nikon epifluorescence microscope equipped with digital camera (Nikon, Yokohama, Japan).

Open software Image J 64, using the MRI Wound Healing Tool was used to quantify.the damaged area.

### Statistical analysis

Three primary cell cultures derived from three different animals were used. Data represent the mean ± SD (or ± range of expression for qRT-PCR) of three biological replicates. Each experiment was repeated two or three times and the data were analysed by Student’s *t*-test or one-way analysis of variance (ANOVA) followed by the Tukey post hoc comparison Test (see the figure legends). Differences of at least *p* < 0.05 were considered significant. Statistical analysis was carried out by using R software (http://www.R-project.org) [56].

## Abbreviations

bFGF: Beta-fibroblast growth factor
CCL20: C-C motif chemokine ligand 20
CSF2: Colony Stimulating Factor 2
CXCL10: C-X-C motif chemokine ligand 10
CXCL-2: C-X-C motif chemokine 2
DPBS: Dulbecco’s phosphate buffered saline
FBS: Fetal bovine serum
GM-CSF: Granulocyte-Macrophage Colony-Stimulating Factor
IFN-γ: Interferon-gamma
IL-10: Interleukin 10
IL-18: Interleukin-18
IL-1α: Interleukin 1-alpha
IL-1β: Interleukin-1beta
IL-2: Interleukin-2
IL-4: Interleukin-4
IL-6: Interleukin-6
IL-8: Inteleukin-8
LPS: Lipopolysaccharide
pAECs: Porcine Aortic Endothelial Cells
pAVPCs: Porcine Aortic Vascular Precursor Cells
PDGF: Platelet Derived Growth Factor
PGM: Pericyte Growth Medium
pVW-MSCs: Porcine Vascular Wall-Mesenchymal Stem Cells
TGF-β: Transforming Growth Factor-beta-1
TLR4: Toll Like Receptor-4
TNF-α: Tumor Necrosis Factor-alpha
VEGF: Vascular Endothelial Growth Factor
